# Lymphomonocytic inflammatory infiltrate with numerous eosinophilic granulocytes in the interstitium in a surviving heart transplant recipient: a case report

**DOI:** 10.3389/fcvm.2024.1341426

**Published:** 2024-04-26

**Authors:** Silvia Placidi, Paola Francalanci, Rachele Adorisio, Katia Girardi, Luciana Vinti, Mario Panebianco, Micol Rebonato, Antonio Amodeo, Giorgia Grutter

**Affiliations:** ^1^ Pediatric Cardiology and Cardiac Surgery Department, Bambino Gesù Children’s Hospital, IRCCS, Rome, Italy; ^2^ Department of Pathology, Bambino Gesù Children’s Hospital, IRCCS, Rome, Italy; ^3^ Heart Failure, Transplant and Mechanical Cardiorespiratory Support Unit, Bambino Gesù Hospital and Research Institute, Rome, Italy; ^4^ Department of Pediatric Hematology and Oncology and of Cell and Gene Therapy, Bambino Gesù Children’s Hospital, IRCCS, Rome, Italy; ^5^ Interventional Cardiology, Bambino Gesù Children’s Hospital, IRCCS, Rome, Italy

**Keywords:** case report, lymphomonocytic inflammatory infiltrate, heart transplantation, endomyocardial biopsy, arteriovenous extracorporeal membrane oxygenation

## Abstract

Findings of eosinophilic and lymphomonocytic inflammatory infiltrates in endomyocardial biopsies (EMBs) may help in myocardial disease diagnosis identification. Eosinophilic myocarditis (EM), a rare condition, is fatal if left untreated and has rarely been described in heart transplant recipients. An extensive work up is necessary to achieve an early etiological diagnosis; however, the underlying cause remains unexplained in nearly one-third of the patients. The cornerstone of treatment is corticosteroids, comprehensive therapy and heart failure management (including advanced mechanical support for fulminant myocarditis). We have described the case of a 17-year-old heart transplant recipient who presented with a cardiogenic shock. He was admitted to our intensive care unit and treated with inotropic drugs, such as milrinone, adrenaline, vasopressin, and levosimendan; the doses of these drugs were in accordance with our internal protocol. The patient underwent cardiac catheterization, coronarography, and right ventricular EMB. EMB revealed inflammatory lymphomonocytic and eosinophil granulocyte infiltrates; thus, steroid therapy was initiated, with complete recovery achieved after 15 days. Performing an early differential diagnosis among eosinophilic infiltration, acute cellular rejection (ACR), and possible chemotherapeutic damage is emerging as an important challenge. To our knowledge, this is the first reported case of a lymphomonocytic inflammatory infiltration with numerous eosinophilic granulocytes in the interstitium in a surviving heart transplant recipient.

## Introduction

More details on eosinophilic infiltrates and lymphomonocytic inflammatory findings in an endomyocardial biopsy (EBM) may be required to allow practitioners to identify and diagnose myocardial diseases, such as acute cellular rejection (ACR) and eosinophilic myocarditis (EM), more accurately. EM is a rare form of myocarditis that is associated with several systemic diseases as well as a high early mortality rate (1). Its underlying causes remain unknown in a relatively large number of cases. Furthermore, the spectrum of clinical presentations associated with EM is remarkably broad; however, the patient prognosis is poor. Given its rarity, EM is often under-recognized. Although a consensus statement on the treatment of EM is lacking, some evidence has supported the use of steroids (2).

## Case

**Figure 1:**
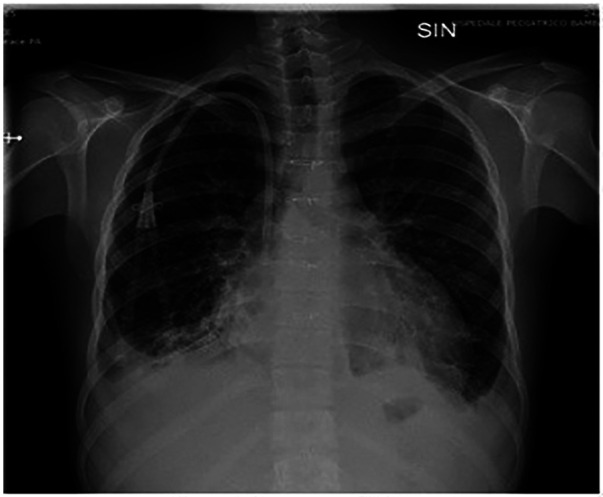
Chest radiograph shows bilateral pleural effusion.

**Figure 2:**
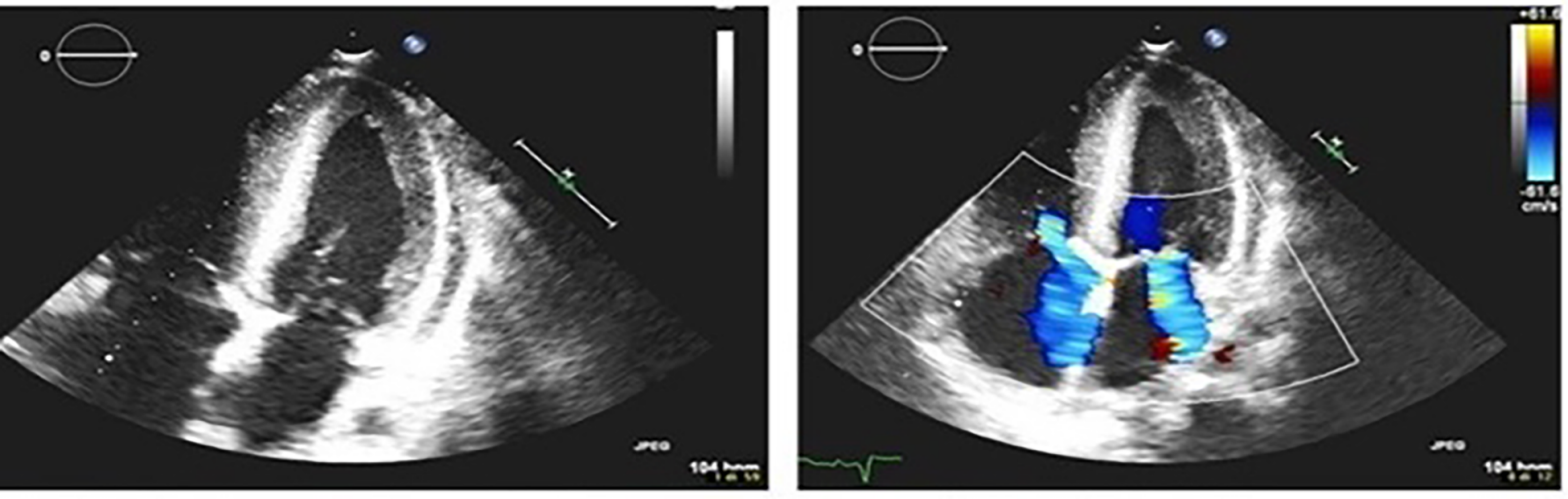
2D-echocardiogram shows moderate mitral regurgitation and thickening of the myocardium.

The patient was a 17-year-old boy who was previously diagnosed with a severe congenital heart disease. At 10 years of age, the patient was listed for heart transplantation and at 12 years underwent heart transplantation. At 16 years of age, the patient was diagnosed with stage 3B Hodgkin's lymphoma post-transplant lymphoproliferative disorder (HL-PTLD) associated with an Epstein–Barr virus (EBV) infection. It is an uncommon PTLD with an unclear prognosis; however, treatment with HL-specific chemotherapy is associated with improved overall survival and event-free survival. The patient received combination chemotherapy with brentuximab vedotin (an anti-CD30 monoclonal antibody) and doxorubicin, vinblastine, and dacarbazinein (BV–AVD). The doses of BV, doxorubicin, vinblastine, and dacarbazinein were 1.2 mg/kg, 25 mg/m^2^, 6 mg/m^2^, and 375 mg/m^2^ (once per day on days 1 and 15), respectively. During the treatment, the mycophenolate mofetile; course was interrupted. Positron Emission Tomography after 2 cycles of BV-AVD revealed a complete metabolic response; the patient received a total of 4 cycles that were well tolerated. No cardiac toxicity was observed during the treatment. At the end of the treatment, disease assessment confirmed a complete metabolic response, and EBV was not detected in the blood. However, 1 month after the last chemotherapy cycle, i.e., at 17 years of age, the patient was admitted to the intensive care unit with symptoms and hemodynamic signs of a cardiogenic shock. Three days prior to this, he had experienced a mild increase in the body temperature (37.5°C) along with decreased energy and poor appetite. Laboratory testing revealed a normal blood cell count with no increase in eosinophils and abnormal cells; furthermore, the C-reactive protein level was normal as well. Troponin I level (3,408.3 pg/ml; high-sensitivity troponin assay) and brain natriuretic peptide level (3,890.1 pg/ml) were clearly elevated, with the creatinine level, EBV CRP copy number, and everolimus level being 1.88 mg/dl, 23,793 copies/ml, and 5 μg/L, respectively. A 12-lead electrocardiogram revealed sinus tachycardia with widespread ST depression. A chest radiograph (Figure 1) revealed bilateral pleural effusion, while a 2D-echocardiogram revealed severe left ventricular dysfunction (ejection fraction: 20%). An altered diastolic function and moderate mitral regurgitation were noted, and a thickening of the myocardium that can be evocative of eosinophilic infiltration (Figure 2). The right ventricular function was also impaired (right ventricular fractional area change, 20%; tricuspid annular plane systolic excursion, 12 mm) with severe tricuspid regurgitation and elevated right ventricular pressure. Thin layers of pericardial effusion and moderate bilateral pleural effusion were also noted. Therapy for acute heart failure was initiated intravenously using inotropic drugs (milrinone, adrenaline, vasopressin, and levosimendan); their doses were determined using an internal protocol. The patient underwent cardiac catheterization, coronarography, and endomyocardial biopsy (EMB) of the right ventricle. Coronarography was negative for obstructive lesions, and no intravascular ultrasound was performed at this time; however, the most recent study revealed a Stanford II coronary artery vasculopathy. Owing to the rapidly worsening hemodynamic stability, arteriovenous extracorporeal membrane oxygenation (ECMO) was initiated. During ECMO, the clinical conditions were critical but more stable.

### Endomyocardial biopsy characteristics

**Figure 3:**
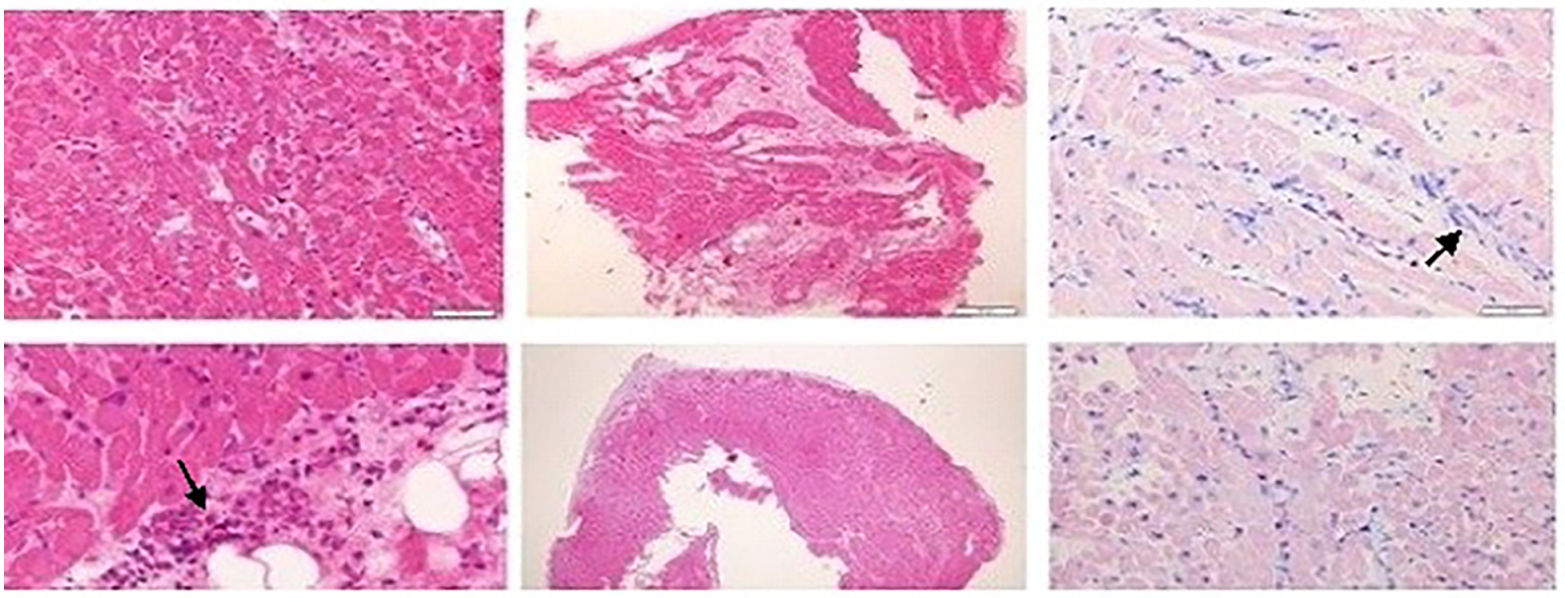
Endomyocardial specimens, shows lymphomonocytic inflammatory infiltrates.

EMB yielded 4 endomyocardial specimens (Figure 3), which were characterized by the presence of lymphomonocytic inflammatory infiltrates (CD3+++, CD8++, CD20−, and CD30−) with numerous eosinophilic granulocytes (partly degranulated) in the interstitium and along the damaged myocellular sarcoplasm. Diffuse interstitial fibrosis was also evident. In 1 fragment, myocyte loss was replaced by a sharply demarcated area of fibrosis with lymphomonocyte infiltration and eosinophilic granulocytes. In the absence of atherosclerotic or inflammatory lesions, the arteries and arterioles showed a preserved wall architecture. Furthermore, focal but dense inflammatory lymphomonocytic infiltration was noted within the endocardium, representing the so-called “quality effect.” Periodic acid-Schiff staining (PAS) and PAS with diastase were negative for fungal hyphae and spores. Polymerase chain reaction assays of the EBM specimens revealed no cardiotropic viruses. Thus, the inflammatory response in the myocardium exhibited features that could have been secondary to ACR, been consistent with EM, or both. Steroid administration (methylprednisolone; 2 mg/kg daily) was initiated intravenously at the beginning and then continued via a nasogastric tube with documented hemodynamic improvement. Biventricular function started to recover on daily echocardiography, and the C-reactive protein and troponin levels soon normalized. After 5 days of high-dose steroid therapy and 7 days of arteriovenous ECMO support, the cardiac function normalized; thus, ECMO was discontinued. However, a severe compartment syndrome of the right inferior limb related to emergency ECMO positioning was observed. Unfortunately, the patient developed a local infection. Acute sepsis and the limbs under the knee were amputated. After 6 months, cardiac magnetic resonance imaging revealed no signs of acute myocarditis, and surveillance EMB was negative for eosinophilic infiltration and signs of rejection. Sixteen months after the acute event, the patient presented with biventricular dysfunction and coronary arteries compromised by Stanford degree IV cardiac allograft vasculopathy. Despite intravenous therapy optimization and considering the high risk of sudden death and lack of other available strategies, the patient was listed for a second heart transplantation. After 2 months on the waiting list, the patient underwent new heart transplantation. No major postoperative complications were noted, and the patient's general condition gradually improved. Two years after, the patient exhibited good hemodynamic and clinical conditions. Histological examination revealed myocytes with polymeric and polymorphic nuclei and a frequently vacuolated cytoplasm. Interstitial fibrosis and occasionally marked and demarcated areas of fibrosis with lymphocytic inflammatory infiltrates (CD3+ and CD8+) were evident in the absence of eosinophils. The coronary arteries in both the initial and peripheral intramyocardial tracts showed marked wall remodelling with intimal hyperplasia, which sometimes caused an almost complete obliteration of the lumen. The pericardium was fibrotic and a site of neovascularization, and had minimal inflammatory infiltrating lymphocytes.

## Discussion

Eosinophilic infiltrates and lymphomonocytic inflammatory findings are helpful in identifying EM or ACR. EM is a rare condition, and its incidence appears to have been underestimated. Eosinophilia has rarely been reported in paediatric heart transplant recipients and has been suggested to play a role in graft rejection (3). In the present case, the patient had recently been treated for HL-PTLD and had been receiving immunosuppressants. Given the patient's clinical history, acute rejection with severe cardiac dysfunction was suspected at presentation. To confirm this hypothesis, an EMB was performed. The indication of biopsy, considering the acute clinical setting and the pediatric age, was given in order to differentiate between eosinophilic infiltration, acute cellular rejection (ACR), and potential damage from chemotherapy, which can be particularly challenging in this complex case and can lead to a different therapeutical approach. No indication for MRI as non-invasive exam was given considering the rapidly worsening hemodynamic stability of the patient. Unexpectedly, the results revealed a cellular infiltrate with predominance of eosinophils, even though the peripheral blood eosinophil count was not elevated and signs of eosinophilic disease were absent. The absence of an increase in the peripheral eosinophil count has been observed in other cases described in the literature (4); this led us to perform a differential diagnosis between eosinophil-rich ACR and EM. EM may have several aetiologies such as the hypereosinophilic syndrome, infections, and immunological disorders. The first 2 were ruled out in our patient after an intensive workup, and we hypothesized that the eosinophilic infiltrate may have been the result of a hyper-immune eosinophilic activation triggered by a delayed-type hypersensitivity reaction to drugs. Hypersensitivity myocarditis is a well-known cardiac manifestation of a delayed-type hypersensitivity response to various drugs; its incidence is elevated in patients on waiting lists for heart transplants (1, 4, 5). However, the specific immune pathways that trigger it remain unknown, especially in the context of heart transplant recipients recently treated with chemotherapy and immunosuppressants, as in our case. We performed a literature review, which revealed only 2 cases of paediatric heart transplant recipients with eosinophilia and eosinophilic myocardial infiltrates; both experienced a sudden death (6, 7). Peripheral blood eosinophilia has been reported in 14.1% of the histologically confirmed cases in a large retrospective series; however, it was absent in our case. We established the mechanism underlying myocardial failure based on EMB findings, which revealed an unexpected myocardial eosinophilic infiltration that guided our treatment strategy. As reported previously (8), EMB remains the gold standard for definitive diagnosis of myocarditis, especially in cases with severe presentation (such as those with fulminant myocarditis). The accurate assessment of EM is limited by difficulties in establishing a diagnosis based on medical history, clinical presentation, and laboratory findings. After heart transplantation, early EMB could be useful in the differential diagnosis of ACR and other possible causes of myocardial injury (such as EM). Our patient presented with a cardiogenic shock and required ECMO support for 7 days. Most data suggest favourable clinical outcomes in patients with fulminant myocarditis supported by percutaneous ECMO (9, 10), as in our patient. Recently, Balthazar et al. (11) reported a case of fulminant EM treated with steroids and mechanical unloading using a percutaneous ventricular assist device in a 42-year-old patient with an unremarkable medical history. These data suggest that alternatives for circulatory support in fulminant myocarditis are determinants of management and depend on the patients' clinical features and the experience of the centre. Our case is also unique because it necessitated the patient to be a candidate for a second transplantation 16 months after the acute event. The patient presented with rapidly worsening and progressive coronary artery vasculopathy after completely recovering from an eosinophilic disease. These conditions require new transplantation, even if the benefits of heart transplantation for any type of myocarditis have been challenged owing to the recurrence risk and high rejection rate. Furthermore, this case emphasizes the need for increasing awareness regarding this rare condition, even in heart transplant recipients, to achieve comprehensive therapy (including advanced therapies for circulatory support). To our knowledge, this is the first report of EM in a surviving paediatric heart transplant recipient. Larger studies and data from international registries are necessary to standardize and validate protocols for the management of this rare disease.

## Data Availability

The raw data supporting the conclusions of this article will be made available by the authors, without undue reservation.
